# An 8-week diet high in cereal fiber and coffee but free of red meat does not improve beta-cell function in patients with type 2 diabetes mellitus: a randomized controlled trial

**DOI:** 10.1186/s12986-018-0324-5

**Published:** 2018-12-29

**Authors:** Yanislava Karusheva, Lejla Kunstein, Alessandra Bierwagen, Bettina Nowotny, Stefan Kabisch, Jan B. Groener, Ann Kristin Fleitmann, Christian Herder, Giovanni Pacini, Klaus Strassburger, Hans-Ulrich Häring, Peter P. Nawroth, Andreas F. H. Pfeiffer, Volker Burkart, Karsten Müssig, Michael Roden, Julia Szendroedi

**Affiliations:** 1 Institute for Clinical Diabetology, German Diabetes Center, Leibniz Center for Diabetes Research at Heinrich Heine University Düsseldorf, Auf’m Hennekamp 65, 40225 Düsseldorf, Germany; 2 German Center for Diabetes Research (DZD), München-Neuherberg, Ingolstädter Landstr. 1, 85764 Neuherberg, Germany; 3 Division of Endocrinology and Diabetology, Medical Faculty, Heinrich Heine University Düsseldorf, Düsseldorf, Moorenstr. 5, 40225 Düsseldorf, Germany; 4 Department Clinical Nutrition, German Institute for Nutritional Research (DifE) Potsdam, Bergholz-Rehbrücke, Arthur-Scheunert-Allee 114-1, 14558 Bergholz-Rehbrücke, Germany; 5 Department of Endocrinology, Diabetes and Nutrition, Charité Universitätsmedizin Berlin, Campus Benjamin Franklin, Hindenburgdamm 30, 12203 Berlin, Germany; 6 Department for Internal Medicine I, University Hospital Heidelberg, Im Neuenheimer Feld 410, 69120 Heidelberg, Germany; 7 Metabolic Unit, Institute of Biomedical Engineering, National Research Council, Corso Stati Uniti, 4, 35127 Padova, Italy; 8 Institute for Biometrics and Epidemiology, German Diabetes Center, Leibniz Center for Diabetes Research at Heinrich Heine University Düsseldorf, Auf’m Hennekamp 65, 40225 Düsseldorf, Germany; 9 Department of Internal Medicine, Division of Endocrinology, Diabetology, Vascular Medicine, Nephrology and Clinical Chemistry and Institute of Diabetes Research and Metabolic Diseases, University Hospital Tübingen, Otfried-Müller-Straße 10, 72076 Tübingen, Germany

**Keywords:** Calorie restriction, Coffee, Dietary fiber, Red meat, Beta-cell function

## Abstract

**Background:**

Higher dietary intake of fibers and coffee, but lower red meat intake is associated with reduced risk for type 2 diabetes in epidemiological studies. We hypothesized that a calorie-restricted diet, which is high in fiber and coffee, but free of red meat, improves beta-cell function in patients with T2D.

**Methods:**

In a randomized parallel-group pilot trial, obese type 2 diabetes patients were randomly allocated to consume either a diet high in cereal fiber and coffee, but free of red meat (*n* = 17) (L-RISK) or a diet low in fiber, free of coffee but high in red meat (*n* = 20) (H-RISK) for 8 weeks. Insulin secretion was assessed from glucagon stimulation tests (GST) and mixed-meal tolerance tests (MMTT) before and after dietary intervention.

**Results:**

Both diets resulted in comparable reduction of fasting concentrations of insulin (H-RISK -28% vs. L-RISK -32%, **both** *p* < 0.01), C-peptide (H-RISK -26% vs. L-RISK -30%, **both** *p* < 0.01) and blood glucose (H-RISK -6.8%, *p* < 0.05 vs. L-RISK -10%, *p* < 0.01). Gastric inhibitory peptide (GIP) secretion increased by 24% after 8 weeks in the L-RISK only (*p* < 0.01). However, GST and MMTT showed no differences in insulin secretion after intervention.

**Conclusions:**

Calorie restriction independent of the intake of fiber, coffee or meat failed to improve beta-cell function, but improved GIP secretion in obese patients with type 2 diabetes.

**Trial registration:**

Registration at Clinicaltrials.gov, Identifier number: NCT01409330, Registered 4 August 2011 – Retrospectively registered.

**Electronic supplementary material:**

The online version of this article (10.1186/s12986-018-0324-5) contains supplementary material, which is available to authorized users.

## Background

Epidemiological studies provided evidence that high fiber, high coffee intake or reduced red meat consumption may delay the onset of type 2 diabetes (T2D) and have beneficial effects on mechanisms underlying its pathogenesis [1–4]. Among others, these dietary components were used to calculate the probability of developing T2D in the European Prospective Investigation into Cancer and Nutrition (EPIC)-Potsdam study [5]. Protein- and fiber-rich products, alongside with caffeic acid and others were shown to enhance insulin secretion, whereas restriction from red meat proved to be a risk-reducing factor for the development of T2D [6–8]. The addition of fiber to a proinflammatory high-fat high-calorie meal had beneficial anti-inflammatory and metabolic effects [9]. Moreover, a high-fiber diet for 16 weeks decreased concentrations of fasting glucose [10], but also acute intake attenuated hyperglycemia [11].

Despite the mostly consistent epidemiological data, intervention studies revealed more diverse results. Indeed, acute coffee consumption shows variable health effects from those achieved by long-term consumption. One single cup of coffee aggravated postprandial glucose excursion in healthy individuals and in patients with T2D [12], whereas regular coffee intake did not influence glucose homeostasis despite its anti-inflammatory effects [13]. Moreover, the EPIC-Potsdam revealed an association between higher red meat consumption and increased risk for T2D [5]. Habitual excess meat intake can cause inflammatory responses as well as oxidative stress [14–17]. In a randomized controlled study, patients with T2D, who abstained from red meat consumption over 4 weeks, showed an increased proportion of serum polyunsaturated fatty acids, which may have a favorable effect on endothelial function, coronary artery disease and albuminuria [18]. Positive associations between red meat consumption and the risk of T2D was evident for both processed and unprocessed preparations [19]. Elevated postprandial amino acid concentrations stimulate insulin secretion without affecting glycaemia [20]. However, diets high in protein from animal or plant sources reduced hepatic fat, hepatic necroinflammation and insulin resistance [21]. Moderately supplementing meat protein with soy protein resulted in improvement of insulin sensitivity as well as total and LDL cholesterol [22]. While these studies tested the effects of single dietary modifications on metabolism, we recently reported on the comparison of two low-energy diets differing in coffee, fiber as well as red meat intake [23, 24]. Both diets equally improved insulin sensitivity and cardiac vagal function in relation to improved oxidative glucose utilization, but failed to affect insulin secretion during an intravenous glucose tolerance test in obese patients with T2D. This test reflects only the glucose-dependent component of insulin secretion under intravenous rather than oral glucose loading conditions, thereby excluding the role of glucagon, incretins and combined effects of other nutrients on in vivo beta-cell function [25]. We hypothesized that a calorie-restricted diet, high in fiber and coffee, but free of red meat, according to the German Diabetes Risk Score (GDRS), improves beta-cell function as assessed by insulin secretion in patients with T2D. To investigate the hypothesis we used both glucagon stimulation and mixed-meal tolerance tests which are established methods for evaluating beta-cell secretory capacity in T2D [26].

## Methods

### Patients and study design

This study was performed in a subgroup of participants of a randomized controlled parallel group trial [23, 24], who underwent two additional tests for beta-cell function on two different days spaced by 8 weeks. Type 2 diabetes patients (18–69 years of age, body mass index (BMI) ≥30 kg/m^2^, known diabetes duration ≤5 years), treated by lifestyle changes and/or with metformin and/or acarbose were included. Exclusion criteria comprised HbA1c > 75 mmol/mol (9.0%), diabetes types other than T2D and acute or chronic diseases including inflammatory diseases or cancer. Patients taking any medication affecting the immune system or insulin sensitivity, other than metformin, were also excluded.

The details of the protocol have been reported elsewhere [23]. A total of 37 obese patients with T2D completed this trial (Additional file 1: Figure S1). A time line of all experiments is given in Additional file 2: Figure S2. The primary endpoint was the M-value derived from the hyperinsulinemic euglycemic clamp test to assess whole-body insulin sensitivity. Based on previously reported data, a mean M-value of 3.8 ± 1.7 mg*kg^− 1^ *min^− 1^ was expected prior to the start of the intervention. For every percentage increase of the M-value, the ratio between the percentage increase of the M-value in the L-RISK group and the percentage increase in the H-RISK group was calculated. Assuming a 20% increase in L-RISK and 16% increase in H-RISK gives a ratio of 1.2 with a conservative estimate of a coefficient of variation of 0.3 and an intraindividual correlation of 0.7. This calculation yields a statistical power of 91%. In order to ensure sufficient numbers of patients per group, we recruited 29/30 persons per group (Additional file 1: Figure S1). The given number of analysed participants of *n* = 15–19 (H-RISK) und *n* = 13–16 (L-RISK) allows detecting moderate effect sizes (Cohen’s d = 0.8) for baseline and 8 week-follow-up differences of measures of beta-cell function from MMT with a power of at least 80%. Thus, the present study was sufficiently powered to detect changes in beta-cell function.

The participants were either assigned to a diet low in fiber (≤10 g/day), coffee-free and high in red meat (≥150 g/day) (H-RISK, *n* = 20) or to a diet high in cereal fiber from wheat and rye (100 g of wholegrain crispread and 250–300 g of wheat/rye wholegrain bread) and fresh-brewed coffee (≥5 cups/day containing 7–8 g coffee powder each, the standard size of coffee cups is 125–150 ml in Europe [5]) and free of red meat (L-RISK, *n* = 17). All participants gave written informed consent before inclusion in the study. The study was performed according to the Declaration of Helsinki, approved by the ethics committee of the Medical Faculty of Heinrich Heine University Düsseldorf and registered at clinicaltrials.gov (Identifier number: NCT01409330).

### Dietary monitoring

Before the start of the intervention, the participants documented their nutritional behavior. During the intervention, all participants received individually calculated daily nutritional protocols, which they had to follow and were asked to document any changes. They had to return the completed sheets to monitor compliance. During the intervention, individually documented food intake was used to assess the participants’ adherence to the study protocol and to ensure consistency of food intake.

### Mixed-meal tolerance test

After overnight fasting for 12 h, participants ingested a standardized liquid meal (237 ml Boost® High Protein, Nestle HealthCare Nutrition, Inc., Florham Park, NJ, USA) containing 33 g carbohydrates, 6 g fat and 15 g protein within 5 min starting at zero time. Blood samples were taken at min − 15, 0, + 30, + 60, + 90, + 120 and + 180 for measurements of glucose, insulin and C-peptide levels to calculate incremental areas under the curve (iAUC), using the trapezoidal rule after subtracting the basal (fasting) values. Insulinogenic index (IGI) was calculated for assessing beta-cell function from the ratio of the difference between insulin levels at baseline and at 30 min to the same difference for glucose levels [27]. The mean basal values of blood glucose and insulin have been previously reported [23].

### Glucagon stimulation test

After overnight fasting for 12 h, blood samples were obtained for measurements of fasting glucose, insulin and C-peptide levels. At zero time, a bolus of 1 mg glucagon (GlucaGen; Novo Nordisk, Mainz, Germany) was injected intravenously and a second blood sample was obtained at min + 6 for measurements of insulin and C-peptide levels [26]. The difference between C-peptide and insulin concentrations between 0 min and 6 min was used to assess glucagon-stimulated C-peptide and insulin secretion capacities (ΔC-peptide and Δinsulin, respectively) [28].

### Laboratory analyses

Serum samples were analyzed as described [26]. Briefly, blood glucose concentration was measured by the hexokinase method (EPOS 5060 analyzer; Eppendorf, Hamburg, Germany). Serum C-peptide and insulin were measured by radioimmunoassay (intra-assay coefficient of variation (CV) for all, 1%; interassay CV, 6–7% and 5–9%, respectively; Millipore, St. Charles, MO, USA) [29]. Glucagon-like peptide 1 (GLP-1) and gastric inhibitor peptide (GIP) were measured using ELISA (GLP-1: interassay CV, 10%; TECOmedical, Sissach, Switzerland; GIP: interassay CV, 12%; Millipore) [29]. Other parameters of clinical chemistry (total cholesterol, low-density lipoprotein (LDL), high-density lipoprotein (HDL) and triglycerides (TG)) as well as liver enzymes were measured on a Cobas c311 analyzer (Roche, Diagnostics, Mannheim, Germany) [26].

### Statistical analyses

The values are shown as mean ± standard error of the mean (SEM). Assuming that the two experimental samples follow Gaussian distribution, statistical significance of differences was assessed with the two-tailed t-test. The effects of both diets on blood glucose and incretin levels and on parameters reflecting insulin secretion were analyzed by repeated measurement two-way ANOVA to compare diet-induced changes between groups as well as effects of time, diet and time - diet interaction. Analyses adjusted for BMI, weight loss and medication were performed to exclude these as confounding factors.

## Results

### Anthropometry

Patients in both H-RISK and L-RISK groups did not differ in age, body mass index, glycemic control and lipidemia and had comparable blood glucose-lowering medications [23].

### Dietary composition and body weight

At baseline, all participants of both groups had comparable calorie, macronutrient, red meat, coffee and cereal fiber intake. All participants adhered to the nutritional protocols as evident by a 6.9-fold higher intake of cereal fiber in the L-RISK group (*p* < 0.0001), a 1.3-fold higher intake of red meat and the absence of coffee consumption in the H-RISK group compared to the L-RISK group (both *p* < 0.0001). As a result of the dietary advice, participants of both groups consumed less total energy and fat, but more carbohydrates and proteins during the intervention [23].

### Mixed-meal-stimulated beta-cell function

**Fig. 1:**
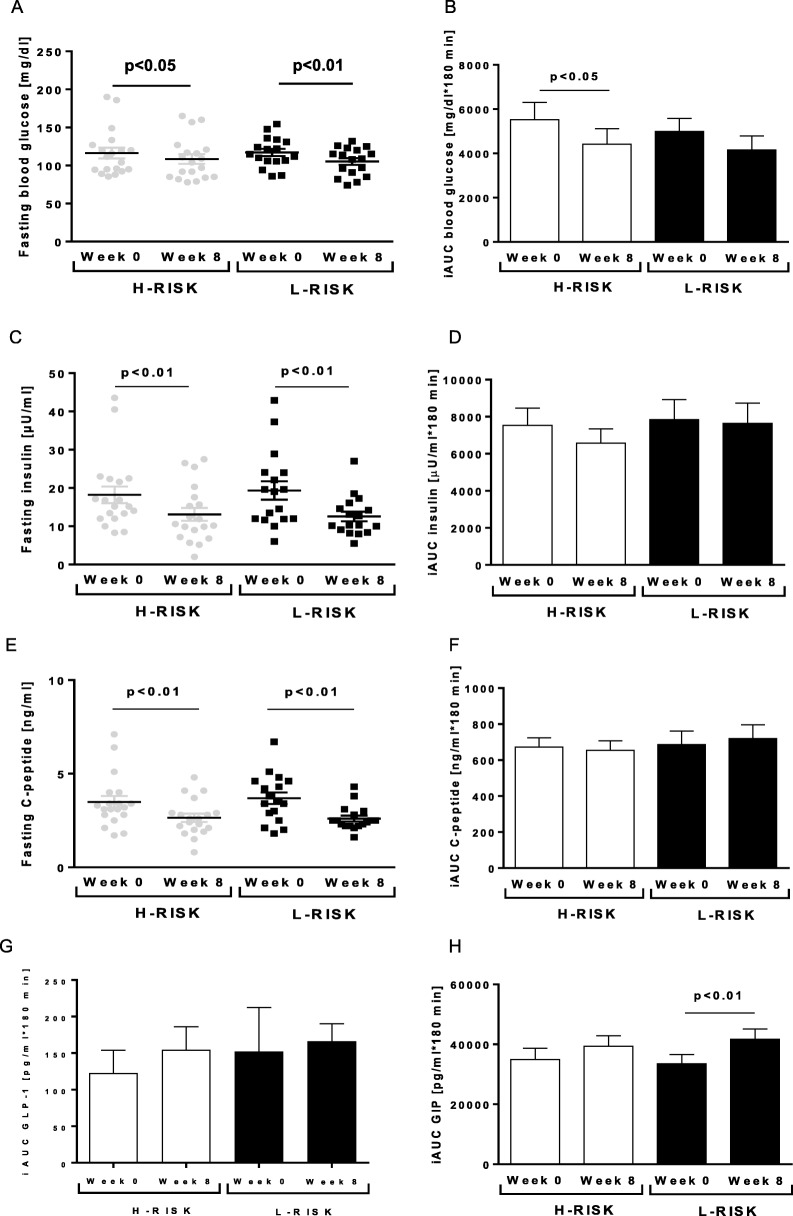
Fasting concentrations of blood glucose (**a**), insulin (**c**), C-peptide (**e**), as well as mixed-meal-induced beta-cell function (means±SEM) from incremental areas under the respective concentration-time curves (iAUC; **b**, **d**, **f**) before and after dietary intervention in a subgroup of participants of the H-RISK group (*n* = 19) and L-RISK groups (*n* = 16). Glucagon-like peptide (GLP-1) (**g**) and gastric inhibitor peptide (GIP) (**h**) from incremental areas under the respective concentration-time curves

At baseline, fasting glucose, insulin and C-peptide (Fig. 1a, c, e) as well as GLP-1 and GIP were similar between both groups. Fasting blood glucose levels measured on the day of the MMTT decreased by 10% (− 11.9 ± 2.8 mg/dl, *p* < 0.01) in the L-RISK group and by 6.8% (− 7.9 ± 2.9 mg/dl, *p* < 0.05) in the H-RISK group (Fig. 1a). Previously reported blood glucose levels were measured on the day of the hyperinsulinemic euglycemic clamp test (HEC), as insulin sensitivity was the primary endpoint [23]. Fasting blood glucose levels assessed before MMTT were not different compared to those measured before HEC (*p* > 0.05 for both groups). Fasting insulin concentration decreased by 28% (− 5.1 ± 1.4 μU/ml, *p* < 0.01) in the H-RISK group and by 32% (− 6.8 ± 1.9 μU/ml, *p* < 0.01) in the L-RISK group (Fig. 1c). Fasting C-peptide concentration decreased by 26% (− 0.8 ± 0.2 ng/ml, *p* < 0.01) in the H-RISK group and by 30% (− 1.0 ± 0.3 ng/ml, *p* < 0.01) in the L-RISK group (Fig. 1e). There were no differences of the changes in fasting glucose and hormone concentrations between both groups. IGI remained unchanged in the H-RISK group before (138.5 ± 15.0) and after (141.2 ± 24.9) as well as in the L-RISK group before (176.0 ± 20.0) and after (206.6 ± 43.2) dietary intervention, showing no change in beta-cell function.

At baseline, iAUC for glucose, insulin, C-peptide, GLP-1 and GIP (Fig. 1b, d, f, g, h) were similar between both groups. After dietary intervention, iAUC for glucose decreased by 20% (− 1109 ± 507 mg/dl*180 min, *p* < 0.05) only in the H-RISK group (Fig. 1b). The iAUC for insulin and C-peptide (Fig. 1d, f) neither changed during L-RISK nor during H-RISK diets. The iAUC for GIP increased only after the L-RISK diet (+ 8152 ± 1993 pg/ml*180 min, *p* < 0.01) (Fig. 1h), whereas the iAUC for GLP-1 remained unchanged after both diets. Further statistical analyses of MMTT revealed no differences in the changes of all parameters from week 0 to week 8 between the H-RISK and L-RISK groups. After adjustments for BMI, weight loss and medication, the results of the analyses remained virtually unchanged.

### Glucagon-stimulated beta-cell function

**Fig. 2:**
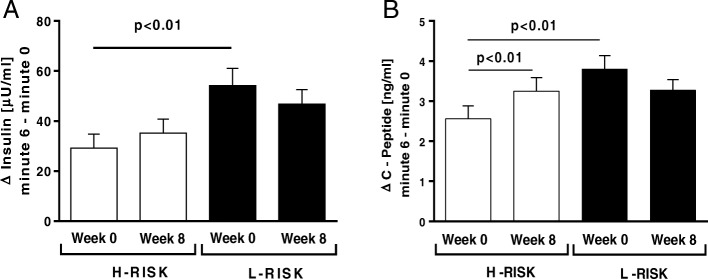
Glucagon-stimulated beta-cell function (means±SEM) from Δinsulin (**a**) and ΔC-peptide (**b**) before and after dietary intervention in a subgroup of participants of the H-RISK (*n* = 15) and L-RISK groups (*n* = 13)

In the L-RISK group, compared to the H-RISK group, Δinsulin and ΔC-peptide concentrations were higher before the intervention (both *p* < 0.01) (Fig. 2a, b). In the H-RISK group, ∆ C-peptide concentration increased by 27% (*p* < 0.01). No changes of Δ insulin were observed in either group. After further adjustments for BMI, weight loss and medication, the results of the analyses remained virtually unchanged.

## Discussion

Eight-week calorie restricted diets, differing in the intake of cereal fibers, coffee and red meat, both failed to improve mixed-meal- or glucagon-stimulated beta-cell function, but decreased fasting insulinemia in obese patients with near-normoglycemic T2D. Both EPIC and the dietary-based diabetes-risk score (DDS) [30] weighted positively low-fat dairy, fiber and coffee and negatively red meat. To design our dietary intervention, we used dietary components established as individual markers of high/low risk of T2D, but not previously used in this very combination. We cannot exclude the possibility of interaction between single dietary components. This dietary multimodal intervention, however, neither evaluated single dietary components nor focused on effects of macronutrients’ components.

In healthy humans, high-fiber diet was found to enhance insulin secretion, indicating improvement of beta-cell function [6]. It is speculated that fiber-rich products contain specific compounds such as trace minerals and phenolic compounds enhancing the acute phase of insulin secretion. In fact, previous studies showed that high-fiber intake lowers the risk of type 2 diabetes [31] and improves glycemic control in patients with overt type 2 diabetes [4]. However, patients of these studies featured mostly diabetes duration of > 5 years and markedly impaired beta-cell function. In the present study, the absence of any effect of modulating fiber intake on insulin secretion may be due to the rather preserved beta-cell function, which probably cannot be further improved by mild to moderate dietary interventions. Nevertheless, we applied an inclusion criterion for known diabetes duration ≤5 years to keep the study group homogenous and the pancreatic insulin production preserved. Previous studies revealed that patients with T2D for up to 6 years duration are more likely to reach a remission of the disease through a dietary or lifestyle intervention [32, 33] suggesting preserved beta-cell secretion capacity.

However, both fasting insulin and C-peptide decreased most likely corresponding to improved insulin sensitivity in both groups as previously shown [23]. In parallel, both groups showed a reduction of fasting blood glucose levels after 8 weeks of intervention, so both diets lead to reducing hyperglycemia. In the H-RISK group iAUC for glucose decreased by about 20%. Furthermore, Δ C-peptide increased by about 27% in the H-RISK group only. In the absence of any changes of Δ insulin, this finding does not indicate a physiologically relevant change of insulin secretion. Of note, both Δ C-peptide and Δ insulin remained unchanged in the L-RISK group. Results of observational studies support the protective effects of coffee and reduced red meat consumption [3, 34, 35]. However, in the setting of our study in patients with overt T2D, these dietary factors also reduce hyperglycemia, but fail to affect insulin secretion.

Of note, coffee consumption of ≥5 cups/day containing 7–8 g coffee powder each, also failed to affect insulin secretion. This is in line with cross-sectional analyses of 1440 Japanese adults using the homeostatic model assessment as a surrogate of insulin secretion [36] and of 1088 elderly Swedish men, assessing the early insulin response during an oral glucose tolerance test (OGTT) [37].

The absence of any effect of dietary modulation of fibers, coffee and meat on insulin secretion in the present study could be due to the short intervention period. In our study, 8 weeks of dietary intervention did not affect insulin and C-peptide secretion in any of the groups as assessed by MMTT. However, in the L-RISK group there was an increase of GIP concentration during MMTT after dietary intervention. This observation corresponds with the results of a previous study showing a decrease of GIP secretion after a high-fat meal, but a preservation of GIP secretion after a high-fiber meal [38].

The similar decrease in fasting insulin and C-peptide levels suggests a comparable improvement in - particularly hepatic - insulin sensitivity by both H-RISK and L-RISK diets. Our initial study preceding this subgroup analysis found that both diets increase whole body insulin sensitivity, without changes in insulin-mediated suppression of endogenous glucose production as assessed from the hyperinsulinemic euglycemic clamp test, which however was not designed to specifically test hepatic insulin sensitivity [23, 24]. Of note, both diets also lowered the increased hepatic fat content, which is generally linked to hepatic insulin resistance [39]. With regard to higher coffee consumption, the observed improvements in insulin sensitivity, but not insulin secretion are in line with some cross-sectional analyses [36, 37], but not with a randomized crossover-study in 26 healthy humans, who drank one liter coffee every day for 4 weeks [40].

The present study benefits from examining beta-cell function with two independent methods and careful dietary monitoring, but suffers from the limited intervention period of 8 weeks and the small sample sizes. In addition, this study cannot account for potential effects of different preparation and processing of macronutrients. Moreover, a disadvantage of the study was that the baseline insulin and C-peptide secretory capacity was different between the two intervention groups despite randomization. To detect further possible effects particularly on insulin secretion, the comprehensive metabolic phenotyping of a several fold higher number of participants would have been required. In addition, both dietary interventions led to a minor (< 5%) weight loss, which could have nevertheless masked certain diet-specific effects.

## Conclusion

In conclusion, a short-term dietary modification with high cereal fibers and coffee, but free of red meat does not improve beta-cell function compared to a diet low in fibers, lacking coffee and high in red meat. Any reduction of hyperglycemia by both diets is not due to changes in insulin secretion.

## Additional files

Additional file 1: **Figure S1.** Flow diagram of participants’ recruitment. (PDF 203 kb)

Additional file 2: **Figure S2.** Time line of study protocol. (PDF 261 kb)

## Abbreviations

AGEs: Advanced glycation end products
BMI: Body mass index
GIP: Gastric inhibitory peptide
GLP-1: Glucagon-like peptide 1
GST: Glucagon stimulation test
HEC: H-RISK, high-risk diet low in fiber, free of coffee and high in red meat
iAUC: Incremental area under the curve
IGI: Insulinogenic index
LDL: Low-density-lipoprotein cholesterol
L-RISK: Low-risk diet high in fiber, coffee (≥5 cups/day) and free of red meat
MMTT: Mixed-meal tolerance test
OGTT: Oral glucose tolerance test
TMAO: Trimethylamine N-oxide
