# Supplementary figures and images for: An 8-week diet high in cereal fiber and coffee but free of red meat does not improve beta-cell function in patients with type 2 diabetes mellitus: a randomized controlled trial
Source: Nutr Metab (Lond). 2018 Dec 29;15:90. doi: 10.1186/s12986-018-0324-5 (PMC6311026; doi:10.1186/s12986-018-0324-5)

**Figure S1. Flow diagram of participants' recruitment**

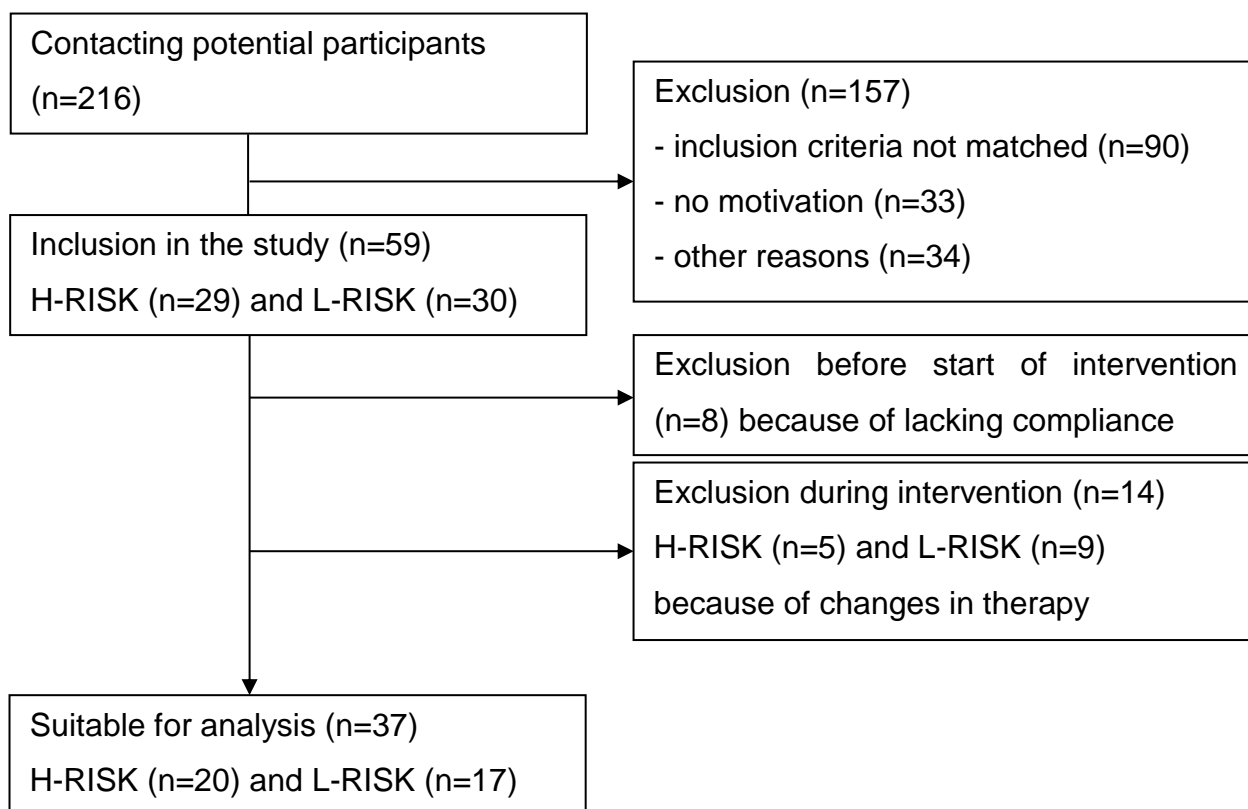

Supplement: Supplementary file 1 — Figure S1. Flow diagram of participants’ recruitment. (PDF 203 kb) [file 12986_2018_324_MOESM1_ESM.pdf]
