# Supplementary material for: An 8-week diet high in cereal fiber and coffee but free of red meat does not improve beta-cell function in patients with type 2 diabetes mellitus: a randomized controlled trial
Source: Nutr Metab (Lond). 2018 Dec 29;15:90. doi: 10.1186/s12986-018-0324-5 (PMC6311026; doi:10.1186/s12986-018-0324-5)
Supplement: Supplementary file 2 — Figure S2. Time line of study protocol. (PDF 261 kb) [file 12986_2018_324_MOESM2_ESM.pdf]

Figure S2. Time line of study protocol

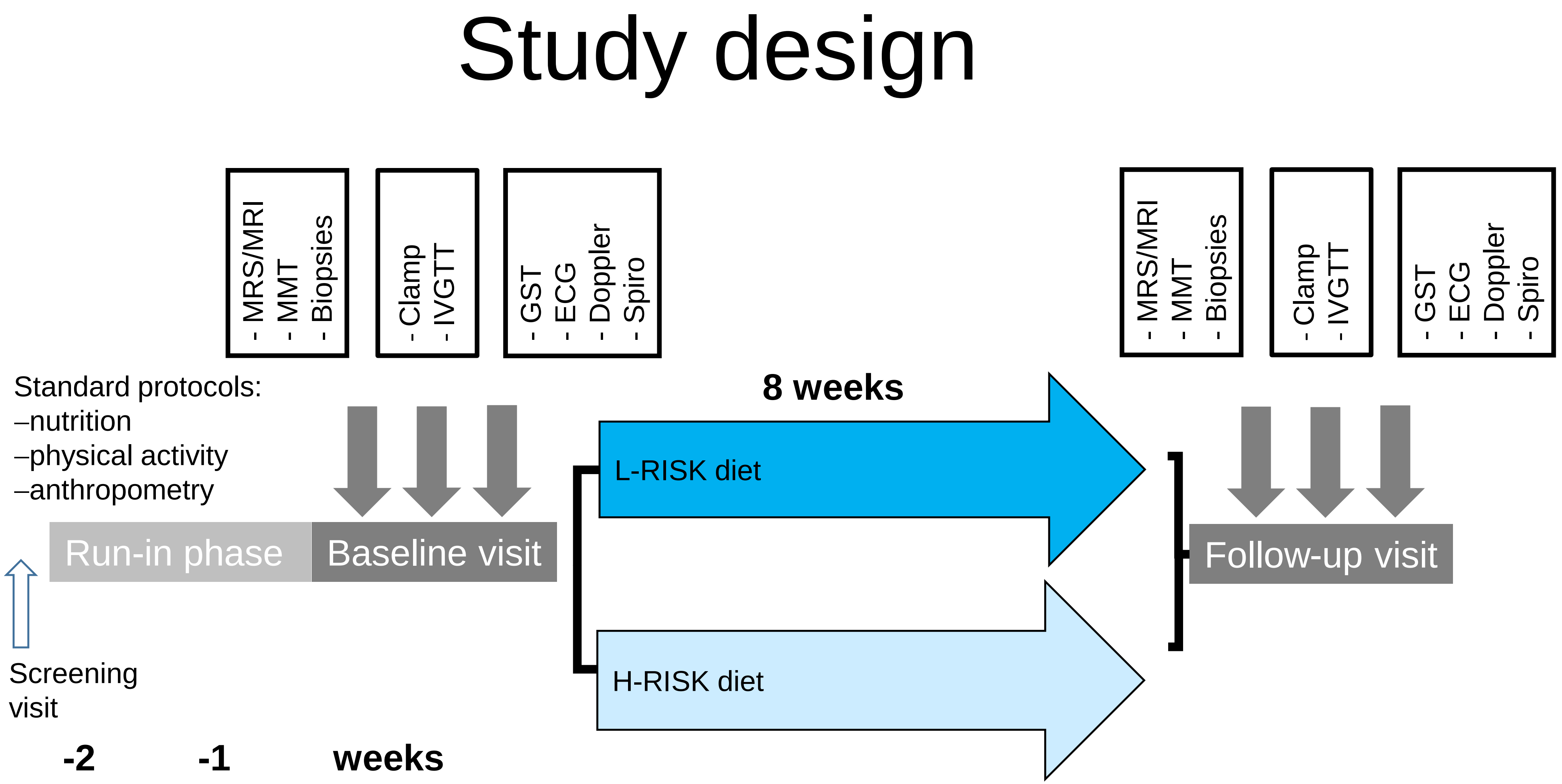

MRS: magnetic resonance spectroscopy
MRI: magnetic resonance imaging
IVGTT: intravenous glucose tolerance test
GST: glucagon stimulation test
ECG: electrocardiography
Spiro: spiroergometry
